# Comparison of donkey and stallion semen preservation: effects of egg yolk plasma, seminal plasma and extenders on sperm motility, MALDI profiles and fertility

**DOI:** 10.1530/RAF-26-0059

**Published:** 2026-09-25

**Authors:** Angélique Petit, Richard Eloy, Valérie Labas, Fabrice Reigner, Amandine Gesbert, Antoine Crampon, Philippe Barrière, Thierry Blard, Yvan Gaude, Xavier Druart

**Affiliations:** ^1^ INRAE, CNRS, Université de Tours, PRC, Nouzilly, France; ^2^ INRAE, Université de Tours, CHU de Tours, Plateforme PIXANIM, Nouzilly, France; ^3^ INRAE, UEPAO, Centre de Recherche de Tours, Nouzilly, France

**Keywords:** donkey, stallion, cooled semen, commercial extenders, egg yolk plasma, seminal plasma, deep intrauterine insemination, fertility

## Abstract

**Abstract:**

The conservation of donkey breeds at risk of extinction requires the development and optimisation of semen preservation and artificial insemination strategies aimed at improving reproductive efficiency, increasing genetic diversity and supporting sustainable population management. The effects of commercial extenders (INRA96, BotuSemen Gold, Beyond), seminal plasma (SP) removal and egg yolk plasma (EYP; 0–10%) supplementation on jack semen stored at 4°C for 24–48 h were evaluated, using stallion semen as a reference. Sperm motility was assessed by computer-assisted sperm analysis (CASA), whereas intact-cell MALDI-TOF mass spectrometry (ICM-MS) was used to characterise peptidoform and proteoform profiles. Fertility was evaluated following low-volume deep intrauterine insemination. Among the preservation strategies tested, the milk protein-based extender INRA96, SP removal and supplementation with 5% EYP were the most effective at preserving sperm motility during storage. After 24 h at 4°C, EYP-supplemented semen exhibited higher total and progressive motility and achieved a conception rate of 35%, compared with 5% for semen stored without EYP (fresh semen: 45%). These functional improvements were accompanied by dynamic remodelling of the sperm proteome, with EYP primarily mitigating cooling-induced molecular alterations rather than altering the basal sperm proteome in both jacks and stallions. The birth of four Grand Noir du Berry (GNB) foals from semen stored for 24 h provided *in vivo* validation of this preservation strategy and highlighted its potential to support assisted reproduction and the management of genetic diversity in donkey populations.

**Lay summary:**

The conservation of endangered donkey breeds relies on maintaining genetic diversity and improving reproductive success, particularly in populations with a limited number of breeding animals. This study aimed to optimise the preservation of donkey semen by evaluating different commercial extenders, as well as the effects of seminal plasma removal and egg yolk plasma supplementation during cooling, building on previous findings in the stallion. The results showed that supplementation of a milk-based extender with 5% egg yolk plasma improved sperm motility and viability after 24 h of storage at 4°C and partially limited alterations in protein profiles associated with cellular ageing. This improvement translated into a conception rate of 35%, compared with only 5% without egg yolk plasma, approaching that achieved with fresh semen (45%). The birth of four Grand Noir du Berry foals from semen stored for 24 h provides *in vivo* validation of this approach and opens new opportunities for assisted reproduction programmes.

## Introduction

The domestic donkey is a species whose population continues to decline, threatening the survival of local breeds such as the Grand Noir du Berry (GNB). Preserving genetic diversity has therefore become a major priority, requiring the establishment and long-term maintenance of cryobanks. However, the low fertility rate obtained with frozen donkey semen does not ensure the renewal of the species (Oliveira *et al.* 2006, Vidament *et al.* 2009). Cryopreservation is characterised by a drastic decrease in temperature, accompanied by cellular dehydration and the formation of ice crystals. These stressful conditions lead to structural and molecular alterations, including changes in membrane permeability, reduced sperm metabolism, increased production of reactive oxygen species (ROS) and DNA fragmentation, which reduce sperm viability and fertilisation capacity (Yánez-Ortiz *et al.* 2022). In contrast, cooled semen maintains acceptable viability for several days when stored under appropriate conditions (Clulow & Gibb 2022, Wiebke *et al.* 2022), making this approach particularly advantageous for semen transport and short-term preservation. This technique also represents an attractive option for breeders aiming to optimise the fertility of their jennies while minimising the costs and time associated with transport.

The tolerance of spermatozoa to low temperatures is largely determined by the biochemical properties of the plasma membrane, particularly the cholesterol-to-phospholipid ratio and the proportion of polyunsaturated fatty acids (PUFAs), which regulate membrane fluidity, structural stability and susceptibility to oxidative stress (Gobato *et al.* 2022). During cooling, phospholipids undergo a phase transition that reduces membrane fluidity and restricts lipid and protein movements (Mocé *et al.* 2010). In stallions, this transition occurs at approximately 20.7°C, and spermatozoa are particularly susceptible to cold shock between 19 and 8°C (Parks & Lynch 1992). This process is influenced by both the cooling rate and final storage temperature (Moran *et al.* 1992). Consequently, a controlled cooling rate of approximately −0.3°C/min is recommended to minimise membrane damage (Varner *et al.* 1988, Santos *et al.* 1995). In jacks, the exact phase transition temperature remains unknown. However, available data indicate that sperm membranes are enriched in PUFAs, which may increase susceptibility to lipid peroxidation during cooling (Wood *et al.* 2016, Prasinou *et al.* 2022, Chaves *et al.* 2023). In addition, donkey spermatozoa exhibit distinct motility patterns and metabolic characteristics compared with stallions, potentially reflecting species-specific differences in mitochondrial function, energy metabolism and the antioxidant capacity of seminal plasma (SP) (Miró *et al.* 2005, Davila *et al.* 2016, Papas *et al.* 2019, Gacem *et al.* 2021).

Despite the close phylogenetic relationship between jacks and stallions, and the growing number of studies characterising the proteome (Swegen *et al.* 2015, Gaitskell-Phillips *et al.* 2022, Talluri *et al.* 2022, Costa *et al.* 2024), metabolome (Castiglione Morelli *et al.* 2021, Bazzano *et al.* 2023) and lipidome (Wood *et al.* 2016, Prasinou *et al.* 2022) of SP and spermatozoa, comparative studies between the two species remain scarce (Ren *et al.* 2024, Wen *et al.* 2025). Nevertheless, emerging evidence has highlighted significant metabolic differences, with stallion SP containing higher levels of lactate, formate and fumarate, whereas jack SP was enriched in myo-inositol as well as amino acids and derivatives such as alanine, phenylalanine, aspartate, glutamate, leucine, isoleucine, valine, taurine, hypotaurine and carnitine (Catalán *et al.* 2023). In addition to these metabolic differences, marked variations have also been reported in the protein composition of SP, with jack SP containing four to ten times more protein and other compounds, such as calcium, lipids and phosphorus, than stallion SP (Talluri *et al.* 2017). These molecular disparities could explain the poor quality of cooled jack semen when preserved in extenders designed for stallions (Vidament *et al.* 2009, Canisso *et al.* 2019, Gobato *et al.* 2022) and confirm that the transfer of biotechnologies from one species to another often results in unsatisfactory outcomes in reproduction (Wildt *et al.* 1995). Several authors have suggested that the poor cooling tolerance of jack sperm may be mitigated either by removal of SP through centrifugation or by supplementation of milk-based extenders with lipids (Rota *et al.* 2008, Di Palma *et al.* 2020, Segabinazzi *et al.* 2021, Gobato *et al.* 2022).

Egg yolk plasma (EYP), obtained after centrifugation and removal of the granular components, contains low-density lipoproteins (LDLs) that interact with sperm membranes, stabilising lipid architecture while reducing viscosity and lipid overload (Anton 2013). Compared with whole egg yolk, EYP can also be sterilised by gamma irradiation (Pillet *et al.* 2011). Artificial phospholipid vesicles, such as liposomes, have also been proposed, although their production remains more costly (Pillet *et al.* 2012; Eterpi *et al.* 2022).

The main objective of this study was to optimise the cooling protocol for jack semen in order to preserve sperm viability and fertility. To achieve this objective, three experimental approaches were conducted using jack and stallion semen: i) comparison of three commercial extenders, ii) evaluation of EYP supplementation and SP removal, and iii) assessment of fertility through artificial insemination (AI) performed at the tip of the uterine horn.

## Materials and methods

### Animals

GNB donkeys (five jacks from 5 to 21 years and 14 jennies from 4 to 16 years) and five Welsh stallions (from 6 to 14 years old) were raised under conventional conditions, in accordance with industry practices, at the equine experiment unit of INRAE (https://eng-pao.isc.inrae.fr, UEPAO, Nouzilly, France). All animal procedures were conducted in accordance with current European legislation (EU Directive 2010/63/EU), approved by the Ethics Committee for Animal Experimentation of Val de Loire (C2EA 19) and authorised by the French Ministry (APAFIS No. 43222-2023042717366637).

### Semen processing

Prior to the experimental period, jacks and stallions were collected several times to standardise semen quality and reduce the potential influence of previously stored epididymal spermatozoa. Semen was collected using a Colorado-model artificial vagina, with either a dummy mount or a mare/jenny depending on the male. Each ejaculate was immediately filtered through a gauze compress to remove the gel fraction and debris. The appearance, colour and gel-free volume of semen were recorded. Sperm concentration and quality were determined using a CASA (IVOS® II, Hamilton Thorne Biosciences, USA). Kinematic parameters related to total motility (TM, %) included average velocity (VAP, μm/s), curvilinear velocity (VCL, μm/s), straight-line velocity (VSL, μm/s), as well as linearity (LIN, %), straightness (STR, %), lateral head displacement amplitude (ALH, μm) and beat cross frequency (BCF, Hz). Progressive motility (PM, %) was defined by the criteria VAP > 40 μm/s and STR > 80%. Measurements were performed using 20 μm calibrated slides (CellVision Technologies, Netherlands, ref. CV 1020-4cv) for greater standardisation of analyses. The analyses were performed on an average of eight fields of view (60 frames per second).

### Experiment 1: comparison of semen extenders and EYP supplementation

#### Preparation of EYP

Unfertilised eggs were broken to separate the white from the yolk. Egg yolks were carefully rolled on filter paper to remove albumen and chalaza adhering to the vitelline membrane. The vitelline membrane was pierced with a scalpel and the yolk collected. The yolk was diluted (1:1) with an equal volume of sodium chloride (NaCl, 0.17 M). The solution was equilibrated at 4°C for 1 h with magnetic stirring and then centrifuged at 10,000 ***g*** for 45 min. The supernatant was collected, centrifuged a second time to remove any trace of granule contamination and stored at −20°C.

#### Semen dilution in commercial extenders and storage conditions

Semen was diluted at 35°C in INRA96 (IMV Technologies, France, ref. 016441), BotuSemen Gold (Botupharma, Brazil, ref. BTSGW-100) and Beyond (Minitube, Germany, ref. 13570/0100) to achieve a final sperm concentration of 20 × 10^6^ spermatozoa/mL (Varner *et al.* 1987, Di Palma *et al.* 2020). INRA96 (pH: 7 ± 0.1 and osmolarity: 310 ± 20) contains caseins, buffers, salts, sugars, ultra-pure water, antibiotics (Penicillin 27 mg/L–Gentamicin 76 mg/L) and a fungicide (Amphotericin B). BotuSemen Gold (pH: 6.8–7.2 and osmolarity:L 355–375) contains sodium caseinate, sugars, amino acids, antioxidants, cholesterol and excipients. Beyond is a chemically defined semen extender that does not contain animal by-products. It is a synthetic medium formulated with pharmaceutical-grade ingredients, including antibiotics and a fungicide. All components are purely synthetic, reducing the risk of uterine inflammation. Eppendorf tubes containing 1.4 mL diluted semen were immersed in 100 mL water at 35 ± 0.5°C and then transferred to a cold room at 4°C for slow cooling. The final sample temperature was reached after 6 h of storage. This protocol aimed to reproduce the cooling curve observed in a standard container used for the transport of cooled equine semen (Equitainer^®^). Sperm quality parameters were assessed immediately after collection and dilution (T0) and after 1, 3, 6, 24 and 48 h of storage (T1, T3, T24 andT48).

To analyse the effect of EYP on sperm quality and viability, INRA96 was supplemented with 0, 2.5, 5 and 10% EYP. Extender pH remained stable across all experimental treatments and was not affected by EYP supplementation. The extender had an initial osmolarity of 307 mOsm, consistent with the manufacturer’s specifications, which increased slightly to 315–325 mOsm after EYP addition while remaining within the physiological range.

#### Intact-cell MALDI-TOF mass spectrometry analysis

Semen was diluted to 20 × 10^6^ spermatozoa/mL in INRA96 supplemented with EYP at different concentrations (0, 2.5, 5 and 10%) and stored for 0, 1, 3, 6, 24 and 48 h at 4°C. Each sample was washed three times with 12.4 mL Tris sucrose (TS) buffer (20 mM Tris–HCl, pH 6.8, 245 mM sucrose) and centrifuged in buckets at 2,000 ***g*** for 10 min at 20°C. At the last step, around 50 μL supernatant was left to resuspend the pellet. Sperm concentration was measured with CASA and adjusted to 150,000 spermatozoa/μL with TS buffer. Samples were frozen and stored at −20°C until ICM-MS analysis.

Prior to analysis, pools were created by combining samples from five jacks on the one hand and five stallions on the other. Each pool corresponding to a treatment was then analysed as an individual sample. Onto a MTP Polished 384 MALDI plate (Bruker Daltonics, Germany), 0.5 μL saturated CHCA (α-cyano-4-hydroxycinnamic acid) matrix dissolved in 100% ethanol was spotted. One microlitre cell suspension (around 150,000 sperm/μL) was overlaid with 2 μL saturated CHCA (α-cyano-4-hydroxycinnamic acid) matrix dissolved in 50% acetonitrile/ 50% water in the presence of 0.1% TFA (trifluoroacetic acid) for jacks and 1.5 μL saturated CHCA matrix dissolved in 50% acetonitrile/50% water in the presence of 0.2% TFA for stallions. Fifteen technical replicates were spotted using the sandwich deposition method. The matrix/sample mix was allowed to evaporate at room temperature for 30 min before MALDI-MS analysis. Spectra were acquired with FlexControl software (version 4.0) using a RapifleX MALDI Tissuetyper TOF mass spectrometer (Bruker Daltonics, Germany) equipped with a Smartbeam 3D Nd:YAG (355 nm) laser. The M5 defocus Smartbeam parameter was set at a 30 × 30 μm scan range with a resulting field size of 109 × 109 μm. Spectra were obtained in positive linear ion mode in the *m*/*z* 1,000–30,000 range, at a 5 kHz laser repetition rate, with a sampling rate of 1.25 GS s^–1^, following an automated method (AutoXecute). Ionisation was achieved using a fixed laser power adjusted according to on-sample test shots in order to reach the optimal ionisation threshold. Each spectrum was collected as a sum of 1,000 laser shots in three-shot steps (total of 3,000 spectra) in a random walk on the spot. Each spot was analysed in triplicate. Spectra were accumulated and evaluated during acquisition between 4,200 and 7,000 *m*/*z*, targeting the major peak with R > 750 for jacks and between 6,000 and 7,000 *m*/*z* targeting the major peak with R > 800 for stallions. Peaks were centroid-detected with a signal-to-noise threshold of 3.00 to detect only the intense peaks before being processed with a Savitzky–Golay smoothing algorithm (5 *m*/*z* width, two cycles) and ‘TopHat’ baseline subtraction. The parameters used for spectra acquisition were as follows: ion source 1, 19.96 kV; electrode P2, 1.60 kV; lens, 8.956 kV; and pulsed ion extraction, 200 ns. The ‘Detector check’ function was conducted regularly to maintain a detector gain at appropriate voltages. After each MS analysis, the MTP MALDI plate was cleaned by sonication in 70% ethanol for 30 min, followed by sonication in two baths of water for 30 min each. The MALDI source was cleaned manually using 90% ethanol and allowed to dry at ambient temperature with pressurised air before pumping until 10^−7^ torr. For each new measurement, the instrument was externally calibrated in quadratic mode using a mixture of peptides and proteins: 1 μL CHCA matrix plus 1 μL calibrant solution containing Glu1-fibrinopeptide B, ACTH (fragments 18–39), insulin, ubiquitin, cytochrome C, myoglobin and trypsinogen.

To increase mass accuracy (mass error < 0.05%), internal calibration was performed on a mix of cells and calibrant solution. A lock mass correction, using flexAnalysis 4.0 software (Bruker) and FlexAnalysis Batch Process (Compass 2.0), was applied to all spectra on mass with high peak intensity. For all profiles, the unknown lock mass corresponding to 9,100.023 *m*/*z* was selected for jacks and 7,656.990 *m*/*z* for stallions. For differential analyses, all spectra were exported in mzXML format with FlexAnalysis Batch Process and treated using R software (version 4.1.3). Spectral processing and statistical analyses were performed using in-house software ‘ROMIC’, scripted from the MALDIquant and MALDIquantForeign packages (v1.19.3 and v0.12) of the R software. For each sample, the dataset (*n* = 15) was filtered by correlation analyses to retain only quality spectra. The correlation coefficients were then calculated based on filters applied with iteration rates of 94% for jacks and 92% for stallions by comparing each technical spectrum with all other spectra within the same class. All retained spectra were merged to generate a total average (median) spectrum for each sample.

Various processes, such as baseline subtraction (SNIP at 350 for jacks and TopHat at 3,500 for stallions), smoothing (Savitzky–Golay algorithm at 80 for jacks and 60 for stallions), realignment (prominent peak method) and normalisation (total ion number method, TIC), were applied to the retained spectra. Peaks were detected using a total average spectrum with a signal-to-background noise ratio >1.2 for jacks and >1.5 for stallions. The selected peaks had to be present at a frequency of 50% in the treatments in which they were detected. The accuracy of the acquisitions was determined by calculating the coefficient of variation from the normalised peak intensity values from the technical replicates for each sample.

The processed spectra obtained from EYP alone, INRA96 alone and spermatozoa diluted in INRA96 supplemented with or without 5 or 10% EYP have been added as Supplementary Fig. 1.

### Experiment 2: effect of SP removal and EYP supplementation

#### Washing procedures and sperm processing

For control samples (unwashed), semen was diluted in INRA96 extender to a final concentration of 20 × 10^6^ spermatozoa/mL. For washed samples, SP was removed by simple centrifugation (15 min, 600 ***g***, 20°C) or by single-layer colloid centrifugation (SLC, 20 min, 400 ***g***, 20°C) using EquiPure^®^ (pH: 7.4–7.8 and osmolarity: 300–310, Nidacon, EPB-100). After centrifugation, the pellet was resuspended in INRA96 at a final concentration of 20 × 10^6^ spermatozoa/mL. For these three treatments, the extender was supplemented or not with 5% EYP. Eppendorf tubes containing 1.4 mL diluted semen were immersed in 100 mL water at 20 ± 1°C and then transferred to a cold room at 4°C. Sperm quality parameters were measured after 1, 3, 6, 24 and 48 h of storage.

### Experiment 3: *in vivo* fertility trials

#### Breeding management

Prior to the fertility trials, all jennies underwent a complete reproductive examination performed by a certified veterinarian. Vulvar conformation, as well as vaginal and uterine status, were evaluated. All jennies tested negative for *Taylorella asinigenitalis* upon arrival and remained free of this bacterium throughout the study period (Breuil *et al.* 2026). Individual data – including age, body weight, number of known offspring and endometrial status – are provided in Supplementary Table 1 (see section on the Supplementary materials given at the end of the article).

Ovarian activity and reproductive tract status were monitored by transrectal ultrasonography using an ExaPad ultrasound device equipped with a 7.5 MHz linear probe. When the dominant follicle reached a diameter ≥32 mm (32–42 mm depending on the individual) and oestrus behaviour was observed (frequent urination, immobilisation and chewing), ovulation was induced by subcutaneous administration of a GnRH analogue (Decapeptyl^®^, 0.1 mg, IPSEN, France).

#### Semen preparation

For fresh semen, ejaculates were filtered, diluted at 35°C in INRA96 extender to a final concentration of 20 × 10^6^ spermatozoa/mL and conditioned in 15 mL Falcon tubes (final volume: 14 mL). Samples were equilibrated in a water bath at 20°C for 10 min and subsequently centrifuged (10 min, 600 ***g***) to concentrate spermatozoa. The supernatant was gently removed and the sperm pellet was resuspended in INRA96 to obtain a final concentration of 400 × 10^6^ spermatozoa/mL.

For cooled semen, ejaculates were collected the day before insemination. Semen was diluted in INRA96 extender supplemented or not with 5% EYP, adjusted to 20 × 10^6^ spermatozoa/mL and aliquoted into 15 mL tubes. Samples were immersed in a beaker containing 400 mL water at 35°C and subsequently stored at 4°C for 24 h. Different sample sets were used for the artificial insemination and mass spectrometry analyses, although they were generated under similar conditions and within the same time period.

#### Artificial insemination of jennies

Twenty-four hours after ovulation induction, jennies were inseminated at the tip of the uterine horn ipsilateral to the dominant follicle under rectal guidance. Each insemination dose contained 200 million spermatozoa packaged in a 0.5 mL straw (IMV Technologies, ref. 005569) and was deposited using a round-tipped catheter (IMV Technologies, ref. 019839).

Oestrous cycles in which ovulation occurred later than 60 h after induction were excluded from the study. Pregnancy was diagnosed 14 days after ovulation. Following pregnancy diagnosis or corpus luteum formation, all females received an intramuscular injection of prostaglandin (Enzaprost®, CEVA Santé Animale, France) to induce luteolysis and synchronise the subsequent ovarian cycle. In pregnant jennies, the subsequent oestrous cycle was not considered in order to avoid any potential influence of the previous reproductive status on the results.

Twenty AIs were performed for each semen treatment (fresh semen and cooled semen stored for 24 h with or without 5% EYP) in ten jennies (mean age: 10 years), with one to three 3 oestrous cycles monitored per female.

### Statistical analysis

Statistical analyses were performed using SAS software (version 9.4, SAS Institute Inc., USA). Data on jack and stallion sperm motility during storage at 4°C in extender containing different EYP concentrations were analysed using a linear mixed-effects model (LMM) fitted with the GLIMMIX procedure in SAS. Concentration of EYP, storage time and their two-way interaction were included as fixed effects. Male was included as a random effect to account for the hierarchical structure of the experimental design, while ejaculate nested within male was specified as the subject for repeated measurements over time. A compound symmetry (CS) covariance structure was used to model the within-subject correlation, as it provided the most stable convergence of the covariance parameter estimates. Denominator degrees of freedom for tests of fixed effects were estimated using the Kenward–Roger approximation. Pairwise comparisons were adjusted for multiple testing using Tukey’s honestly significant difference (HSD) test. Statistical significance was set at *P* ≤ 0.05.

The effects of extender, seminal plasma and washing method on sperm motility were analysed using the GLM procedure in SAS. When model residuals were not normally distributed and/or variances were not homogeneous among groups, non-parametric analyses were performed using the Kruskal–Wallis test for comparisons involving more than two groups or the Mann–Whitney test for comparisons between two groups, implemented with the NPAR1WAY procedure. Fertility rates were compared using a chi-square test. A level of significance of 5% (*P* ≤ 0.05) was adopted for all tests.

For mass spectrometry, normality was checked using a Kolmogorov–Smirnov test. Data were analysed with a non-parametric Wilcoxon test to identify changes between two treatments and a Kruskal–Wallis test to identify changes between several treatments. The *m*/*z* ratios were considered differential between the groups with a *P*-value < 0.01. Hierarchical clustering (Spearman correlations), principal component analysis (PCA), 2D peak distribution and volcano plot were performed using the gplots (version 3.0.3) and FactoMineR (version 2.1) packages of the R software. The PCAs were performed with all the masses detected and with the differential *m*/*z* ratios only.

## Results

### Sperm parameters of fresh semen in jacks and stallions

Jack ejaculates had a gel-free volume of 60.2 ± 4.4 mL and a sperm concentration of 165 ± 11.2 × 10^6^ spermatozoa/mL (Table 1), whereas stallion ejaculates had a lower volume (16.1 ± 2.0 mL) and a higher sperm concentration (274 ± 19.3 × 10^6^ spermatozoa/mL). Accordingly, the total number of spermatozoa per ejaculate was approximately two-fold higher in jacks than in stallions (9.9 × 10^9^ vs 4.4 × 10^9^, respectively). Sperm concentration was negatively correlated with ejaculate volume in stallions (*r* = −0.72, *P* < 0.0001) and with age in jacks (*r* = −0.62, *P* < 0.0001). Although TM was similar between species, jacks displayed higher values for PM, VAP, VSL, LIN, STR and BCF compared with stallions, reflecting greater sperm velocity and more linear movement. No significant differences were observed for VCL.

**Table 1:** Comparison of ejaculate characteristics and sperm kinematic parameters between donkeys and stallions immediately after semen collection. Data are shown as mean ± SEM. Means were compared using Student’s *t*-test (*P* ≤ 0.05).

	Jack (*n*=42)	Stallion (*n*=32)
Volume (mL)	60.2 ± 4.4^a^	16.1 ± 2.0^b^
Concentration (M/mL)	165 ± 11.2^b^	274 ± 19.3^a^
TM (%)	84.2 ± 1.3	82.6 ± 1.7
PM (%)	62.1 ± 1.6^a^	52.9 ± 1.9^b^
VAP (μm/s)	131 ± 1.8^a^	124 ± 2.1^b^
VCL (μm/s)	203 ± 3.4	207 ± 5.6
VSL (μm/s)	114 ± 1.7^a^	101 ± 2.3^b^
LIN (%)	58.9 ± 1.1^a^	53.1 ± 1.4^b^
STR (%)	85.9 ± 0.6^a^	81.3 ± 0.7^b^
ALH (μm)	7.5 ± 0.2^b^	8.2 ± 0.3^a^
BCF (Hz)	36.5 ± 0.3^a^	34.0 ± 0.4^b^

^a,b^ Values without a common letter are significantly different.

### Quality of cooled semen and effect of extender

The sperm kinematic parameters measured immediately after dilution and after 24 h of storage at 4°C in the three commercial extenders are presented in Table 2 for jacks and Table 3 for stallions.

**Table 2:** Kinematic measurements in donkey spermatozoa after semen dilution and 24 h of storage at 4°C. Data are shown as mean ± SEM (*n* = 4 ejaculates per condition). Means were compared using one-way ANOVA or Kruskal–Wallis test (*P* ≤ 0.05).

	INRA96	BotuSemen	Beyond
After dilution (T0)			
TM (%)	85.0 ± 2.7	83.3 ± 3.9	84.5 ± 3.5
PM (%)	60.4 ± 4.8	63.4 ± 4.3	62.1 ± 4.0
VAP (μm/s)	133 ± 5.9	135 ± 8.8	140 ± 6.0
VCL (μm/s)	210 ± 13	208 ± 11	211 ± 5.7
VSL (μm/s)	114 ± 6.3	119 ± 7.4	123 ± 6.0
LIN (%)	57.5 ± 3.6	58.1 ± 1.9	58.7 ± 3.1
STR (%)	84.4 ± 1.5	86.9 ± 0.6	86.2 ± 1.2
ALH (μm)	7.7 ± 0.7	7.4 ± 0.4	8.1 ± 0.6
BCF (Hz)	36.5 ± 0.6^b^	40.0 ± 0.8^a^	35.9 ± 1.0^b^
After 24 h of storage (T24)			
TM (%)	43.4 ± 9.6^a^	13.4 ± 2.7^b^	17.7 ± 3.9^b^
PM (%)	23.3 ± 5.9^a^	8.4 ± 2.4^a,b^	2.8 ± 1.0^b^
VAP (μm/s)	121 ± 7.3^a^	90.5 ± 8.9^b^	89.3 ± 5.4^b^
VCL (μm/s)	244 ± 9.3^a^	178 ± 11.8^b^	201 ± 7.8^b^
VSL (μm/s)	96.4 ± 6.6^a^	74.6 ± 9.8^a^	51.7 ± 2.4^b^
LIN (%)	40.2 ± 2.0^a^	42.3 ± 2.7^a^	28.1 ± 0.7^b^
STR (%)	77.8 ± 1.5^a^	80.5 ± 2.5^a^	60.5 ± 0.8^b^
ALH (μm)	9.5 ± 0.4^a^	7.7 ± 0.3^b^	8.7 ± 0.4^a,b^
BCF (Hz)	35.5 ± 0.5^a,b^	33.8 ± 1.9^b^	38.7 ± 0.5^a^

^a,b^ Values without a common letter are significantly different.

**Table 3:** Kinematic measurements in stallion spermatozoa after semen dilution and 24 h of storage at 4°C. Data are shown as mean ± SEM (*n* = 6 ejaculates per condition). Means were compared using one-way ANOVA or Kruskal–Wallis test (*P* ≤ 0.05).

	INRA96	BotuSemen	Beyond
After dilution (T0)			
TM (%)	82.5 ± 4.8	79.4 ± 4.7	82.0 ± 3.7
PM (%)	44.7 ± 4.7	44.9 ± 4.8	46.6 ± 4.2
VAP (μm/s)	121 ± 4.5	118 ± 1.8	124 ± 2.3
VCL (μm/s)	231 ± 14	211 ± 10	203 ± 11
VSL (μm/s)	93.4 ± 3.6	92.4 ± 3.9	96.7 ± 2.9
LIN (%)	43.8 ± 2.7	47.1 ± 3.9	51.5 ± 3.6
STR (%)	76.9 ± 1.3	78.1 ± 2.6	77.9 ± 1.7
ALH (μm)	9.3 ± 0.7	8.3 ± 0.5	8.0 ± 0.5
BCF (Hz)	34.7 ± 0.6	36.4 ± 0.9	34.3 ± 1.3
After 24 h of storage (T24)			
TM (%)	69.4 ± 5.6^a^	63.5 ± 3.8^a,b^	50.3 ± 5.3^b^
PM (%)	40.6 ± 4.7^a^	34.4 ± 2.6^a^	16.7 ± 2.3^b^
VAP (μm/s)	90.7 ± 4.1^b^	111 ± 3.4^a^	118 ± 2.2^a^
VCL (μm/s)	184 ± 6.1^c^	207 ± 5.2^b^	237 ± 7.5^a^
VSL (μm/s)	71.0 ± 4.1^b^	85.8 ± 2.8^a^	77.9 ± 2.0^a,b^
LIN (%)	40.7 ± 1.5^a^	44.0 ± 1.3^a^	35.7 ± 1.4^b^
STR (%)	79.5 ± 1.2^a^	77.5 ± 1.6^a^	66.0 ± 1.0^b^
ALH (μm)	8.2 ± 0.2^b^	8.0 ± 0.2^b^	9.7 ± 0.5^a^
BCF (Hz)	31.3 ± 1.2^b^	36.4 ± 0.6^a^	35.4 ± 0.8^a^

^a,b,c^ Values without a common letter are significantly different.

Immediately after dilution at 35°C (T0), TM was comparable between the two species, with 84 ± 1% in jacks and 81 ± 2% in stallions. However, PM was higher in jacks than in stallions (62 ± 2% vs 45 ± 2%, respectively), independently of the extender used (Tables 2 and 3, T0). In both species, dilution in INRA96, BotuSemen Gold or Beyond extenders prior to cooling did not affect sperm viability or motility parameters, except for higher BCF values in jacks with BotuSemen Gold, suggesting a higher flagellar beat frequency.

After 24 h of storage, TM decreased moderately in stallion semen, whereas jack semen showed a marked decline in all extenders (*P* ≤ 0.05), indicating reduced sperm survival after cooling (Tables 2 and 3, T24). In jack semen, TM and PM were significantly higher with INRA96 than with Beyond or BotuSemen Gold, whereas these parameters were comparable between INRA96 and BotuSemen Gold in stallion semen. Furthermore, jack semen stored in INRA96 exhibited higher ALH (>8 μm) and VCL (>230 μm/s), together with reduced linearity (LIN <50%). A similar pattern was observed in stallion semen stored in Beyond.

### Effect of egg yolk plasma in milk-based extender

Total motility was significantly affected by storage time in both jacks (Fig. 1A) and stallions (Fig. 1B), decreasing between T0 and T24 (*P* < 0.0001). In jacks, no differences were observed among EYP concentrations at T0. However, after 24 h of storage, TM was significantly higher in EYP-supplemented samples, reaching 69 ± 5% with both 5 and 10% EYP, compared with 36 ± 5% in the control without EYP. In stallions, EYP supplementation had no significant effect on TM.

**Figure 1:**
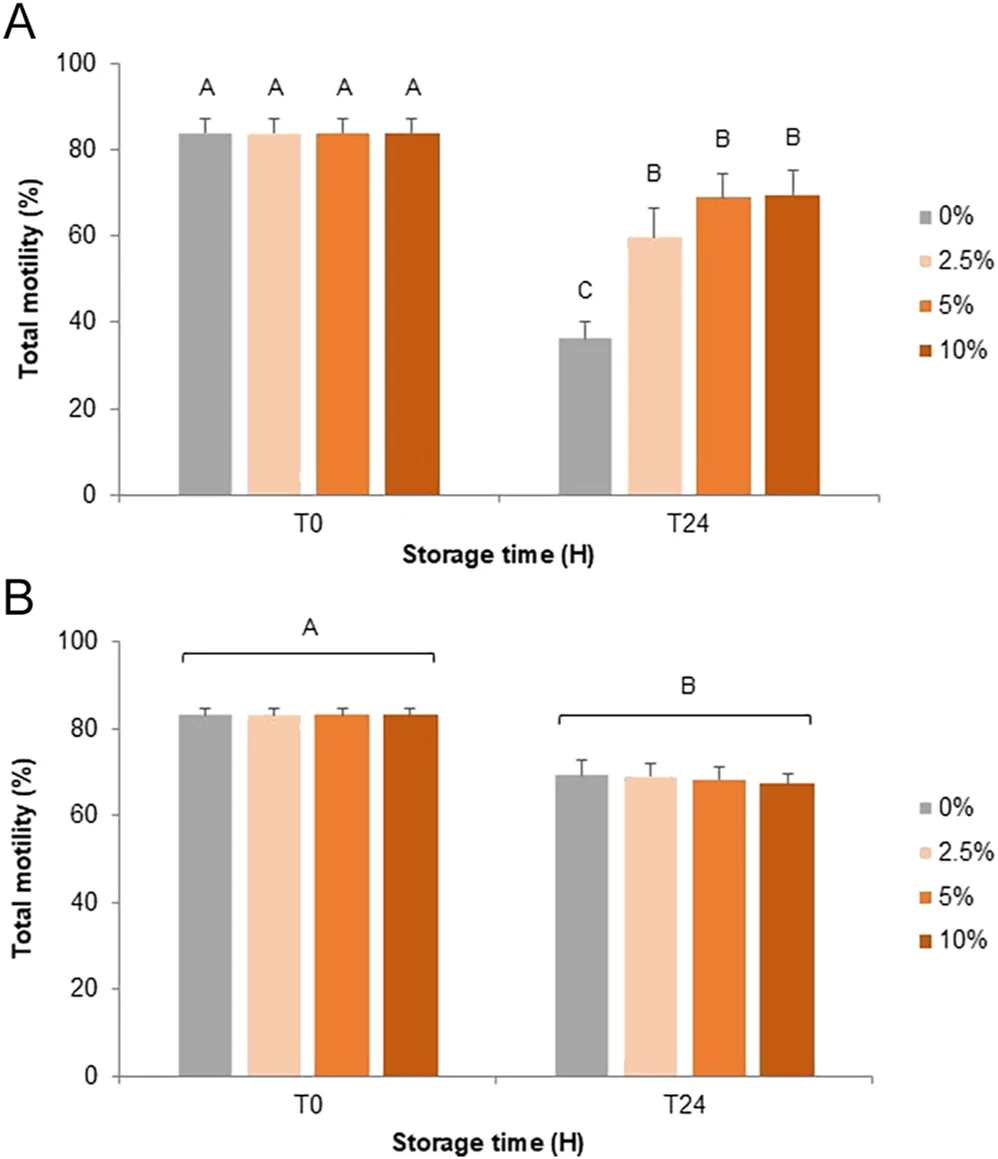
Effect of EYP (0–10%) on total motility during storage at 4°C in jacks (A) and stallions (B). Data are presented as mean ± SEM. Values without a common letter (A, B, C) are significantly different (*P* ≤ 0.05). Semen samples were collected from four jacks (three ejaculates per male; *n* = 12 ejaculates) and five stallions (three ejaculates per male; *n* = 15 ejaculates). EYP, egg yolk plasma; NS, not significant.

In stallions, TM decreased slightly between T0 and T48, whereas in jacks the most pronounced decrease occurred during the first hour of cooling (Fig. 2A). No significant differences were observed between T1 and T3 or between T6 and T24. Overall, TM decreased by 17% in stallions and 61% in jacks over the 48 h storage period. Supplementation of INRA96 with 5% EYP improved jack sperm motility to levels comparable with those observed in stallions (Fig. 2B). In jacks, TM remained relatively stable between T1 and T48 (70 ± 3%), whereas in stallions it declined significantly from T24 onwards.

**Figure 2:**
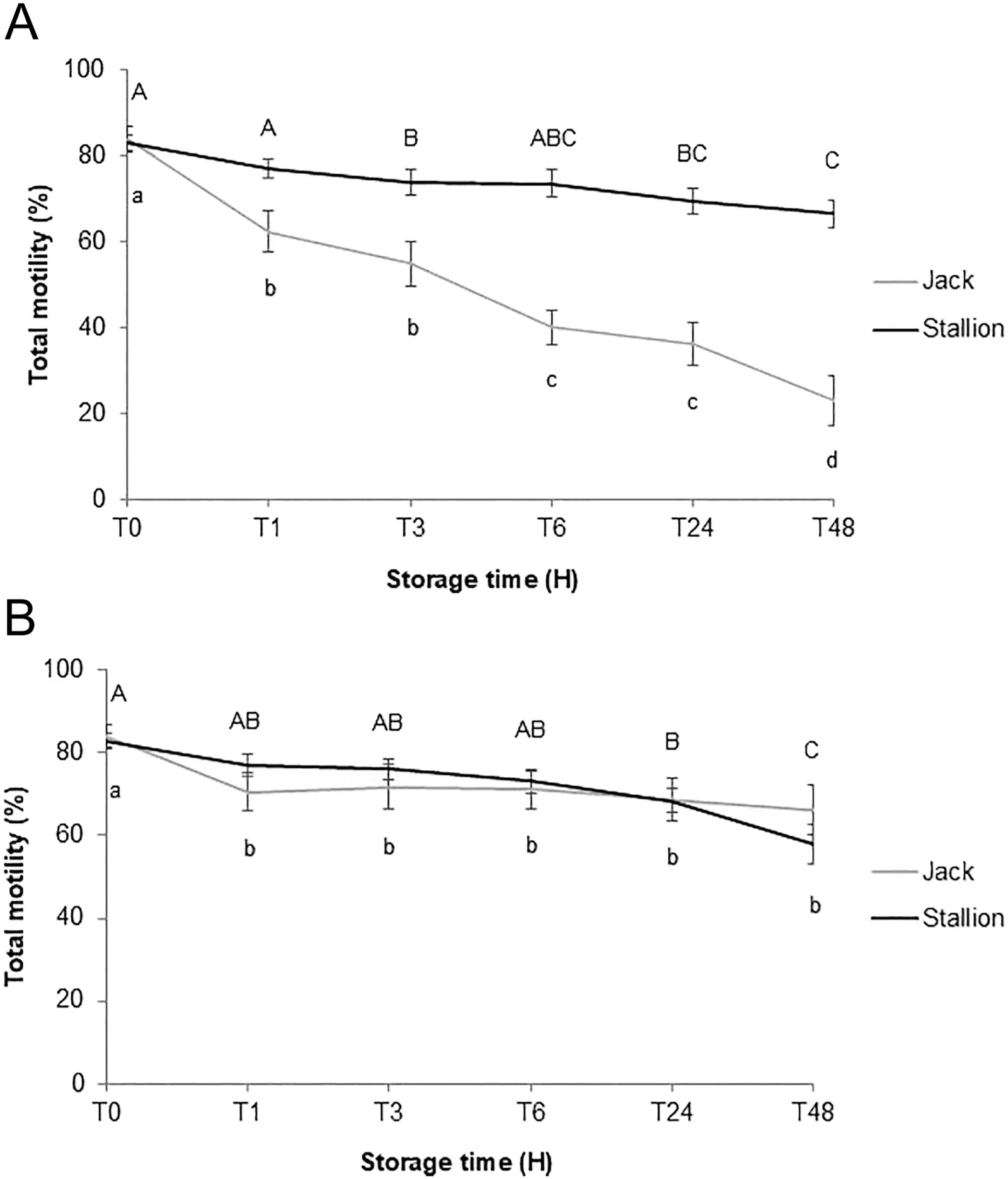
Changes in total sperm motility in jacks and stallions during storage in INRA96 extender, either alone (A) or supplemented with 5% EYP (B). Data are presented as mean ± SEM. Values without a common letter (A, B, C or a, b, c, d) are significantly different (*P* ≤ 0.05). Semen samples were collected from four jacks (three ejaculates per male; *n* = 12 ejaculates) and five stallions (three ejaculates per male; *n* = 15 ejaculates).

### Evolution of protein profiles during storage with and without egg yolk plasma

The PCA of proteoform and peptidoform profiles from semen stored at 4°C, regardless of EYP concentration, is shown in Fig. 3A for jacks and Fig. 3B for stallions.

**Figure 3:**
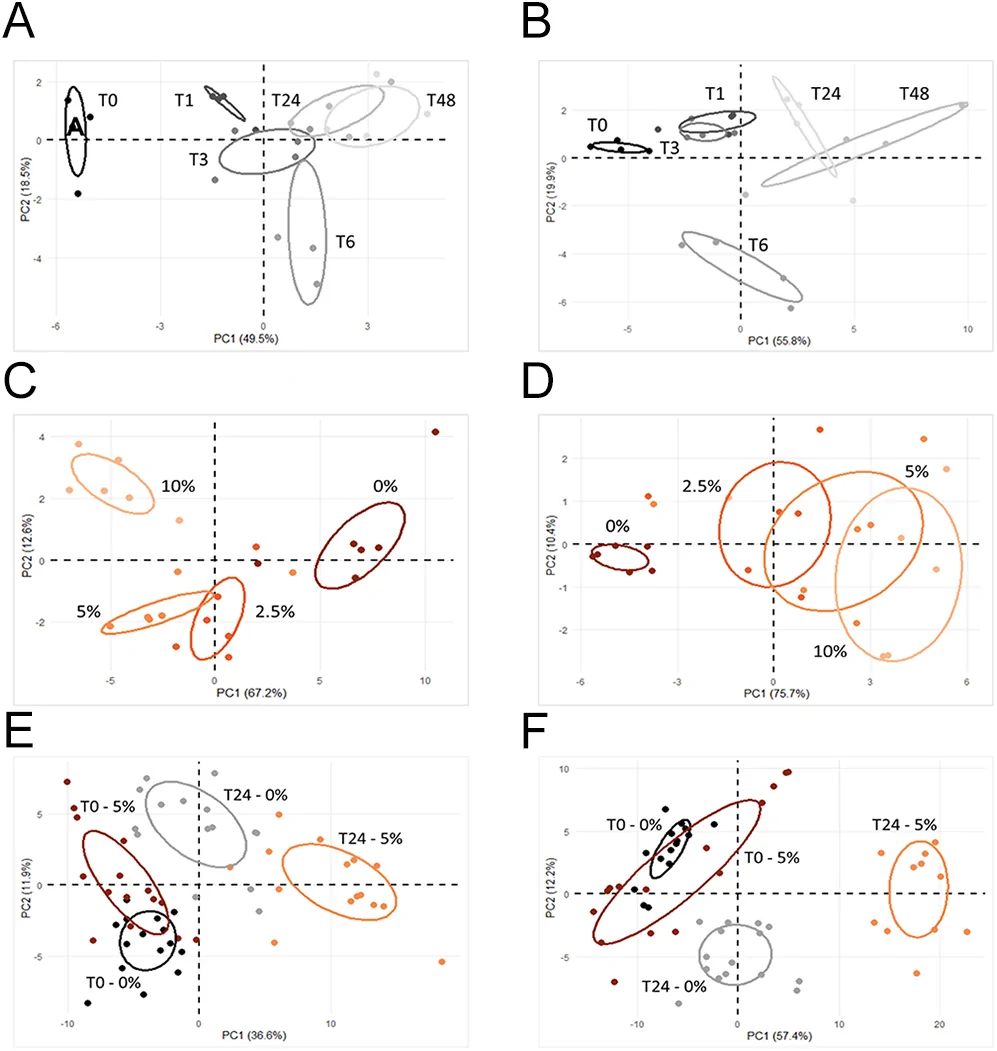
PCA of jack (A) and stallion (B) sperm proteoform and peptidoform profiles according to storage time at 4°C, regardless of EYP concentration. Time points (T0, T1, T3, T6, T24 and T48) are shown using a grey colour gradient. Each point corresponds to the mean profile for one storage time. PCA of jack (C) and stallion (D) sperm proteoform and peptidoform profiles according to EYP concentration, regardless of storage time. EYP concentrations (0, 2.5, 5 and 10%) are shown using an orange colour gradient. Each point corresponds to the mean profile for one EYP concentration. PCA of jack (E) and stallion (F) sperm proteoform and peptidoform profiles according to both storage time and EYP supplementation. Only peaks with *P* < 0.01 were included in the analysis. Colours indicate the experimental groups: T0–0% (black), T0–5% (dark red), T24–0% (grey) and T24–5% (orange). Each point represents one technical replicate (spectrum), and ellipses represent the 50% confidence interval for each group. The percentage of variance explained by PC1 and PC2 is indicated on the corresponding axes.

In jacks, PC1 and PC2 accounted for 68% of the total variance (Fig. 3A). Along the PC1 axis, a pronounced rightward shift was already observed after the first hour of storage (T1), indicating early changes in sperm proteoform and peptidoform profiles. This shift became more marked between T3 and T6, accompanied by a greater dispersion of the samples. Samples collected at later time points clustered together along the PC1 axis, suggesting the establishment of a distinct and stable molecular signature between T24 and T48. In stallions, PC1 and PC2 explained 76% of the total variance (Fig. 3B). Although the T1 and T3 groups partially overlapped, a clearer temporal trajectory was observed than in jacks, with T6 samples showing a marked shift along the PC2 axis.

The PCA of proteoform and peptidoform profiles as a function of EYP concentration, regardless of storage time, is presented in Fig. 3C for jacks and Fig. 3D for stallions.

In jacks, PC1 and PC2 accounted for 80% of the total variance (Fig. 3C). The extreme EYP concentrations (0 and 10%) were clearly separated along the PC1 axis. The intermediate concentrations (2.5 and 5%) partially overlapped but remained distinct from the extreme concentrations on both PC1 and PC2. In stallions, PC1 and PC2 accounted for 86% of the total variance (Fig. 3D). As observed in jacks, an effect of EYP supplementation was observed, with the control (0%) separated from the other concentrations along the PC1 axis. However, the ellipses corresponding to the 2.5, 5 and 10% concentrations showed significant overlap and greater variability in proteoform and peptidoform profiles. Overall, the effect of EYP concentration appeared less pronounced in stallions than in jacks.

Figure 3E and F shows PCA analyses of proteoform and peptidoform profiles from jack and stallion semen, respectively, considering two factors: storage time (T0 vs T24) and EYP concentration (0 vs 5%).

In jacks, PC1 and PC2 accounted for 49% of the total variance (Fig. 3E). Along the PC1 axis, T0 samples were located on the left (negative values), whereas T24 samples were predominantly located on the right (positive values), highlighting the predominant effect of storage at 4°C. PC2 reflected the secondary influence of EYP concentration. The clear separation of the four experimental treatments indicated progressive remodelling of proteoform and peptidoform profiles during storage, which was modulated by the addition of EYP. In stallions, PC1 and PC2 accounted for 70% of the total variance (Fig. 3F). The effect of storage time appeared even more pronounced in stallions than in jacks, with the T0–0%, T0–5% and T24–0% treatments more clearly separated along the PC1 axis, whereas the T24–0% treatment was positioned lower on PC2. Moreover, EYP supplementation increased within-group variability, as illustrated by the particularly broad ellipse of the T0–5% treatment. Finally, the presence of EYP induced a marked shift in the T24–5% treatment, which occupied a distinct area of the PCA and was clearly displaced along the PC1 axis.

In jacks, comparison of T0 and T24 samples in the absence of EYP identified 19 discriminant *m*/*z* signals among the 223 detected (Fig. 4A; *P* < 0.01; fold change ≥ 1.5; AUC ≥ 0.8). In the presence of 5% EYP, the signal intensity of five of these *m*/*z* signals (2,551, 5,521, 6,708, 11,033 and 13,490 *m*/*z*) remained consistently low throughout storage, indicating that EYP markedly attenuated the storage-induced temporal changes in these signals. In stallions, 60 discriminant *m*/*z* signals were identified among the 280 detected using the same statistical criteria (Fig. 4B). For the remaining signals, regardless of species, EYP supplementation was associated either with an increase in signal intensity (e.g. 2,254 *m*/*z* in jacks or 3,830 *m*/*z* in stallions) or with no detectable change (e.g. 2,570 *m*/*z* in jacks or 1,668 *m*/*z* in stallions).

**Figure 4:**
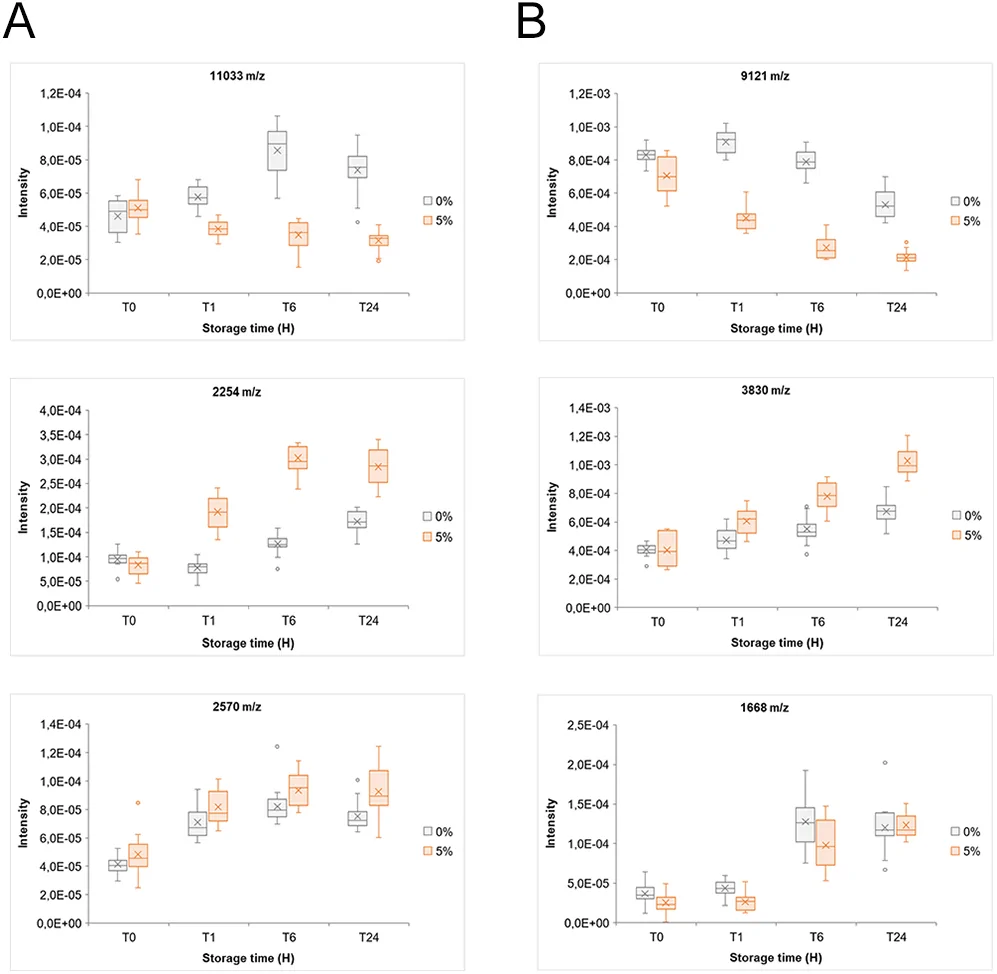
Temporal evolution of the intensity of selected MALDI peaks (2,254–2,570–11,033 *m*/*z* in jacks (A); 1,668–3,830–9,121 *m*/*z* in stallions (B)) according to storage time (T0, T1, T6 and T24) and EYP concentration (0 and 5%). Data are presented as box plots, with crosses indicating mean values. Samples containing 0% EYP are shown in grey, whereas samples supplemented with 5% EYP are shown in orange.

### Effect of seminal plasma removal

For both jacks and stallions, removal of SP did not affect TM immediately after dilution (T0), regardless of the extender used (Fig. 5A). After 24 h of storage at 4°C, centrifugation significantly increased TM in jack semen, whereas no significant effect was observed in stallion semen (Fig. 5B). In jacks, TM was 1.5-, 2.5- and 2.75-fold higher following SP removal than in untreated semen for INRA96, BotuSemen Gold and Beyond, respectively. Among the extenders, INRA96 maintained higher TM (55 ± 6%) than BotuSemen Gold (21 ± 4%) and Beyond (30 ± 6%). No significant interaction was detected between extender and SP removal.

**Figure 5:**
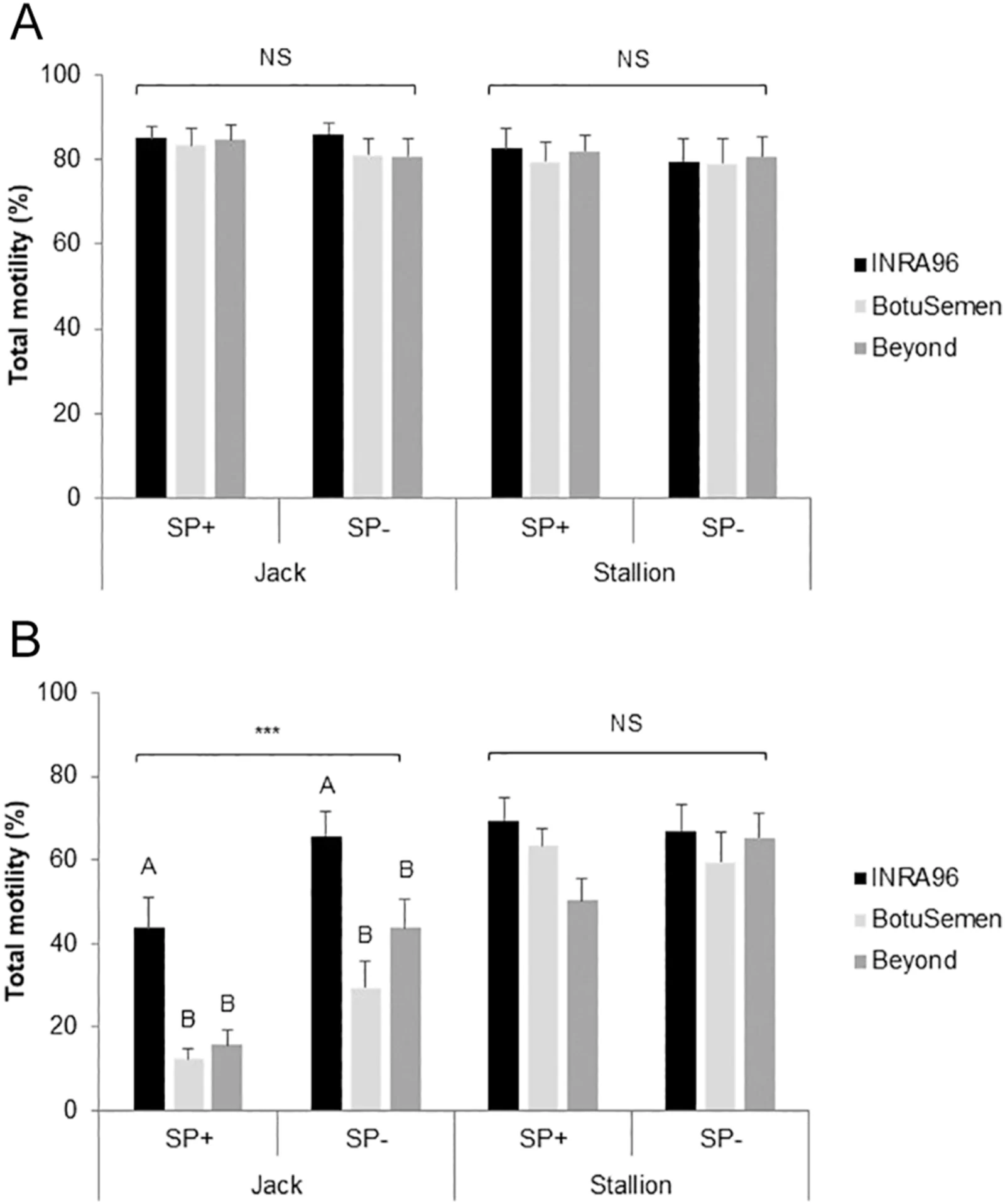
Effect of seminal plasma removal on total motility after semen dilution (A) in the three extenders (INRA96, BotuSemen Gold, Beyond) and after 24 h of storage (B) in jacks and stallions. Data are presented as mean ± SEM. Values without a common letter (A and B) and comparisons with asterisks are significantly different (*P* ≤ 0.05). Semen samples were collected from four jacks and six stallions (one ejaculate per male). SP+, with seminal plasma; SP−, without seminal plasma; NS, not significant.

Total motility following SP removal by simple centrifugation or SLC centrifugation is presented in Fig. 6. In jacks, TM was significantly higher following SP removal by SLC centrifugation compared with the unwashed control (71 ± 7% vs 44 ± 3%, Fig. 6A). Simple centrifugation resulted in intermediate TM values (62 ± 5%), which did not differ significantly from those obtained after SLC treatment.

**Figure 6:**
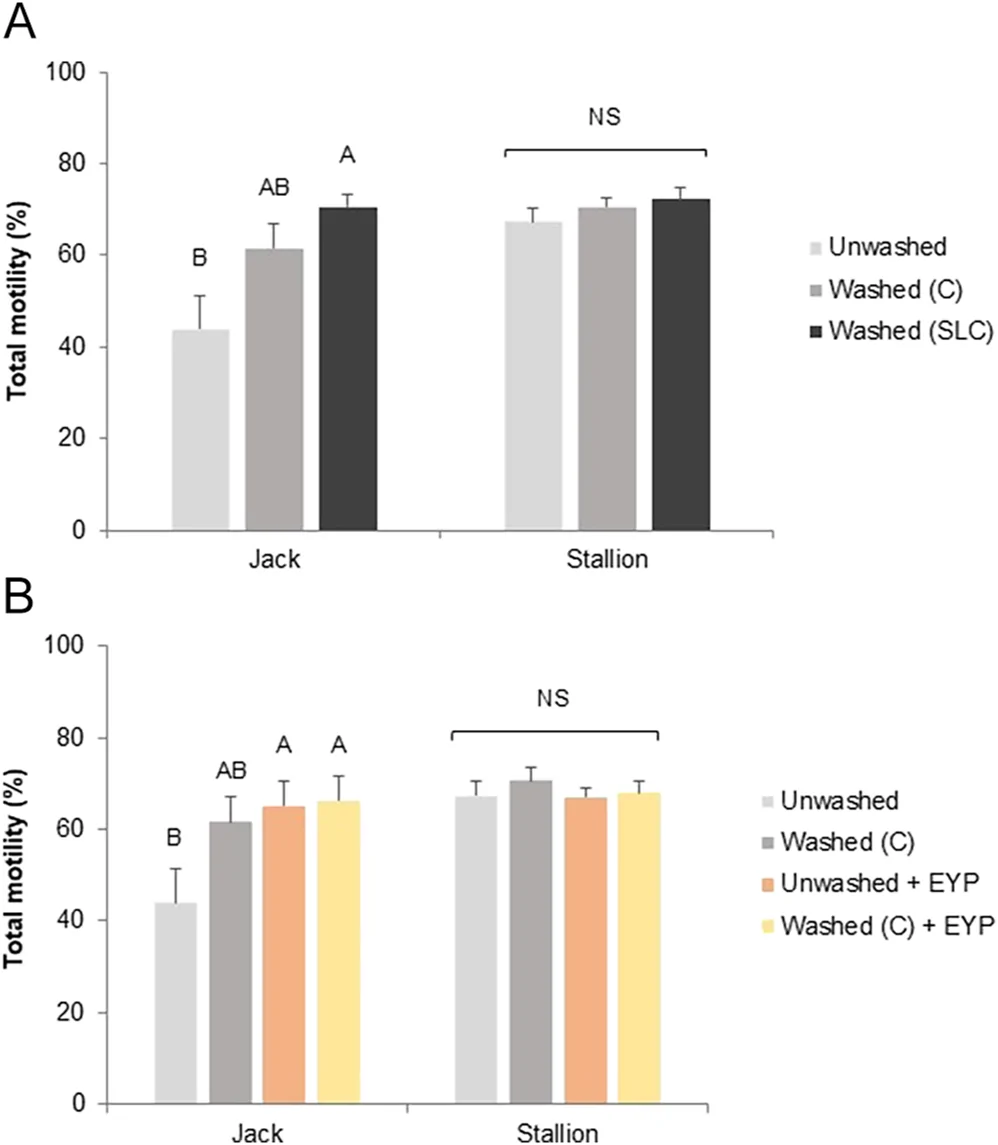
(A) Effect of washing by conventional or colloid centrifugation on total motility after 24 h of storage in jacks and stallions. (B) Effect of seminal plasma removal ± 5% EYP on total motility after 24 h of storage in jacks and stallions. Data are presented as mean ± SEM. Values without a common letter (A and B) are significantly different (*P* ≤ 0.05). Semen samples were collected from four jacks (three ejaculates per male; *n* = 12 ejaculates) and five stallions (three ejaculates per male; *n* = 15 ejaculates). Washed, samples in which seminal plasma was removed; EYP, egg yolk plasma; C, conventional centrifugation; SLC, single-layer centrifugation.

Regardless of the washing method, the addition of 5% EYP did not improve TM (Fig. 6B). The beneficial effect of EYP was observed only in the presence of SP. No differences were observed among treatments in stallions.

### Fertility rates

Figure 7 illustrates the fertility outcomes obtained under different experimental treatments. Deep intrauterine insemination performed with fresh semen resulted in a significantly higher fertility rate (45%, 9/20) compared with semen cooled for 24 h (5%, 1/20). However, supplementation with 5% EYP prior to cooling significantly improved fertility (35%, 7/20). No significant difference was observed between fresh semen and cooled semen supplemented with EYP.

**Figure 7:**
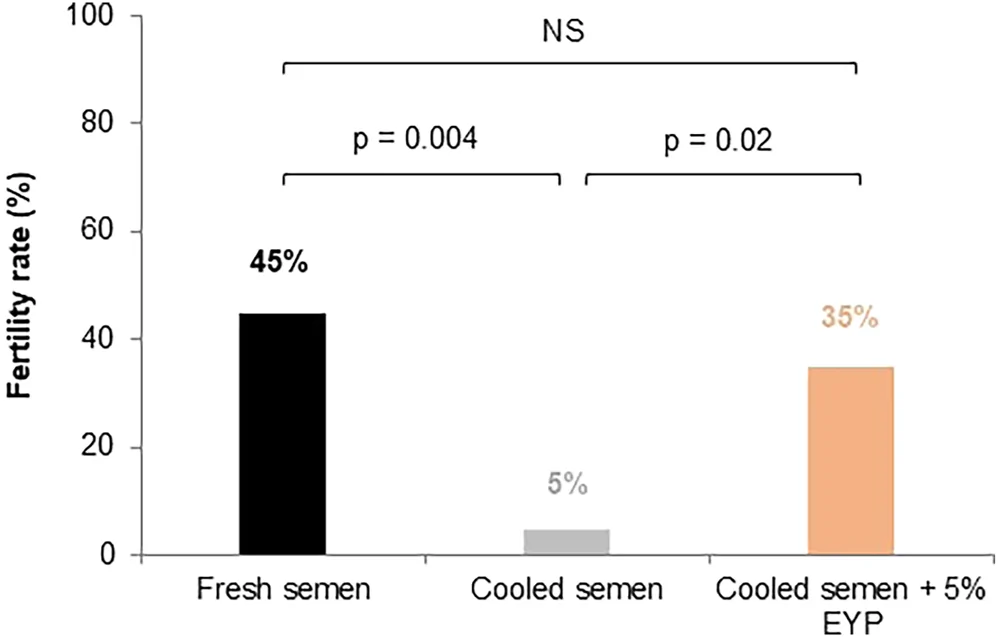
Fertility rates obtained after intrauterine insemination at the tip of the uterine horn using fresh semen or semen cooled for 24 h, with or without 5% EYP. Pregnancy diagnostics were performed on day 14 post-ovulation. A total of 20 inseminations were performed per experimental treatment in 10 jennies, with one to three oestrous cycles evaluated per female. NS, not significant; EYP, egg yolk plasma.

## Discussion

Spermatozoa are generally cooled to temperatures between 4 and 15°C to reduce metabolic activity, prevent premature capacitation and preserve their fertilising potential (Gibb & Aitken 2016). However, the effectiveness of cooling protocols is highly species-dependent and those developed for horses cannot be directly applied to donkeys (Di Palma *et al.* 2020, Gobato *et al.* 2022). In this study, donkey semen was more sensitive to refrigeration at 4°C than stallion semen, with sperm motility and viability decreasing over the 24 h storage period, with the most pronounced decline occurring during the first hour, concomitant with a marked temperature drop from 35 to 17°C. To limit cooling-induced cellular damage in spermatozoa and partially restore fertility after deep intrauterine insemination, the INRA96 extender was supplemented with 5% EYP. This native phosphocaseinate-based commercial extender appears to contribute more effectively to membrane stabilisation, support sperm metabolism and maintain sperm viability and motility during storage at 4°C than BotuSemen Gold and Beyond. Nevertheless, jack semen stored in INRA96 for 24 h exhibited changes in kinematic parameters compared with fresh semen, characterised by increased VCL and ALH and reduced LIN. These modifications reflect a more erratic motility pattern, which may be consistent with hyperactivation-like behaviour or altered kinematics associated with reduced sperm quality, including the possible contribution of a more heterogeneous sperm population (McPartlin *et al.* 2009, Hinrichs & Loux 2012). Based on the mean CASA parameters alone, these two possibilities cannot be clearly distinguished. Hyperactivation is a complex physiological process that typically occurs after ejaculation under conditions promoting capacitation, notably within the female oviduct. Hyperactivated sperm exhibit amplified and non-linear movements that facilitate detachment from the oviduct epithelium, progression through viscous environments and penetration of the zona pellucida (Leemans *et al.* 2019). Although these mechanisms are necessary for successful fertilisation, the associated reduction in sperm lifespan emphasises the importance of optimising insemination timing (Samper 2001).

Beyond functional sperm parameters, molecular approaches may provide additional information on the cellular alterations occurring during semen preservation. In this context, MALDI-TOF MS is particularly well-suited for the rapid and reproducible untargeted profiling of abundant endogenous proteoforms and peptidoforms below 30 kDa. Previous studies have demonstrated its ability to characterise sperm molecular phenotypes associated with epididymal maturation in boars (Pyttel *et al.* 2014, Labas *et al.* 2015, Liu *et al.* 2016), to identify fertility-associated markers in chicken sperm (Soler *et al.* 2016) and to monitor cellular responses to external stressors (Munteanu *et al.* 2014, Chiu *et al.* 2015), supporting its relevance as a proof-of-concept approach for evaluating sperm storage-induced molecular changes.

In this study, PCA revealed that storage time was the main source of variation in sperm proteoform and peptidoform profiles in both species. In jack sperm, T0 samples were clearly separated from all subsequent storage time points, indicating that cooling to 4°C rapidly induced marked molecular changes. In stallion sperm, a more progressive temporal trajectory was observed, with gradual displacement of profiles towards later storage times (T24–T48). Overall, these results highlight time-dependent molecular remodelling during semen preservation, with distinct species-specific trajectories.

EYP supplementation also contributed to variation in sperm molecular profiles, although to a lesser extent than storage time. The absence of a clear dose-dependent trajectory suggests that EYP may induce qualitative rather than linear quantitative changes in sperm molecular profiles. The limited separation between 0 and 5% EYP at T0, compared with the marked divergence observed after 24 h of storage, indicates that EYP primarily mitigates cooling-induced molecular alterations rather than the basal sperm proteome.

From a mechanistic perspective, the observed increase in specific low-molecular-weight signals may reflect the fragmentation of higher-molecular-weight proteins into smaller peptides (<20 kDa), as spermatozoa are transcriptionally and translationally inactive. This interpretation is supported by previous studies demonstrating the proteolytic degradation of multiple sperm proteins through the quantification of the resulting peptide fragments (Labas *et al.* 2015). However, caution is required in the biological interpretation of these molecular signatures, as detected signals may not exclusively originate from sperm proteins. Extender components, particularly milk proteins, may also contribute to the observed profiles through interactions with the sperm membrane during storage. Complementary targeted proteomic analyses will therefore be required to identify the proteins underlying the detected *m*/*z* signals and to distinguish endogenous sperm-derived proteins from exogenous proteins originating from the extender. Previous work in ram semen has shown that egg yolk supplementation at 15% modifies the sperm proteome, including the presence of exogenous proteins such as vitellogenin, apolipoprotein, complement C3 and ovotransferrin, as well as changes in endogenous sperm proteins involved in antioxidant defence and metal ion homeostasis (Pini *et al.* 2018). By comparison, the present study suggests that a lower EYP supplementation range (5–10%) is sufficient for jack semen to achieve optimal preservation without detrimental effects on sperm function. At higher concentrations, EYP may adversely affect sperm function, highlighting the importance of optimising its concentration to maintain sperm quality during storage (Watson 1975, Amirat *et al.* 2004).

At the molecular level, the protective effects of EYP are thought to involve mechanisms similar to those described for milk caseins, potentially involving rapid, specific and saturable interactions between LDLs and Binder of Sperm (BSP) proteins (Foulkes *et al.* 1980, Bergeron *et al.* 2004, Bergeron & Manjunath 2006, Plante *et al.* 2015). SP contains numerous components that support sperm function (e.g. proteins, amino acids, antioxidant enzymes and carbohydrates), but it may also negatively affect sperm survival during cooling (Wang *et al.* 2022). Among these components, BSP proteins play a key role by binding to the sperm membrane and promoting the efflux of cholesterol and phospholipids. The loss of membrane lipids reduces membrane stability and increases sperm susceptibility to cold shock (Drobnis *et al.* 1993). The deleterious effects, which may also be associated with the activity of enzymes such as lipases (Carver & Ball 2002), can be mitigated by high semen dilution ratios (≥3:1) or by removal of SP prior to storage (Jasko *et al.* 1991, Miró *et al.* 2009). In jacks, we showed that SP removal improved total and progressive sperm motility after 24 h at 4°C. Although colloid centrifugation has been reported to selectively retain the most robust spermatozoa with intact DNA, membranes and acrosomes and higher mitochondrial activity (Johannisson *et al.* 2009, Papas *et al.* 2020), no additional benefit was observed in our conditions compared with simple centrifugation. Given the lack of additional benefit and the increased procedural complexity of colloid centrifugation, standard centrifugation represents a simpler and more practical approach for SP removal in jack semen. In contrast, SP removal had no significant effect on stallion sperm, consistent with a previous report describing some stallions as ‘good coolers’ (Brinsko *et al.* 2000). These interspecies differences likely reflect variations in SP composition and sperm–plasma interactions, which may underlie species-specific responses to cooling (Talluri *et al.* 2017).

In jack semen, the beneficial effect of EYP supplementation was observed only when SP was present, suggesting that EYP primarily counteracts SP-induced deleterious effects. After SP removal by centrifugation, no additional cumulative benefit of EYP supplementation was detected. The protective effect of EYP likely results from multiple complementary mechanisms, including membrane stabilisation, modulation of membrane lipid phase transition and interactions with SP proteins, rather than from a single mode of action. To evaluate the *in vivo* relevance of EYP supplementation, AI trials were performed using a 5% EYP concentration, selected based on CASA-derived motility parameters and MALDI-TOF molecular profiling. A fertility rate of 35% was achieved with semen cooled for 24 h with 5% EYP, compared with only 5% without EYP and 45% with fresh semen. The fertility rate achieved with fresh semen was consistent with those previously reported in jennies (40–73%), despite differences in sperm number (200 million vs 400 million to 1 billion), insemination volume (0.5 mL vs 4–15 mL), site of deposition (uterine horn vs uterine body) and number of inseminations before ovulation (single vs multiple) (Vidament *et al.* 2006, De Oliveira *et al.* 2016, Fanelli *et al.* 2019, Fanelli *et al.* 2023). Similarly, cooled semen has been associated with fertility rates of 45–49% following uterine body insemination using 400 million to 1 billion spermatozoa in 4–10 mL (Vidament *et al.* 2009, Yang *et al.* 2021). These findings suggest that EYP improves the fertility of cooled donkey semen and that acceptable fertility can be maintained despite marked reductions in sperm number and insemination volume when semen is deposited closer to the utero-tubal junction.

The fertility rate achieved with fresh semen (45%) may have been influenced, at least in part, by the relatively advanced mean age of the jacks (16 years). In stallions, sperm quality improves from approximately 4 years of age, reaches its peak at around 12 years and begins to decline after 13 years (Abah *et al.* 2023). As in other domestic species, age-related changes in sperm quality are associated with declining epididymal function and progressive testicular degeneration, whereas younger males are primarily affected by immature spermatogenesis (Dowsett & Knott 1996). Although similar age-related changes are likely to occur in donkeys, evidence remains limited. Nevertheless, EYP improved fertility in all jacks included in the study, suggesting that its beneficial effects were maintained despite potential age-related differences in semen quality. Although these findings are encouraging, the use of native EYP in semen extenders remains associated with several practical limitations. As noted by Bustani & Baiee (2021), native EYP-based extenders are subject to variability in composition and carry a potential risk of microbial contamination, which may compromise reproducibility and biosafety. In the present study, eggs were obtained from a single broiler line raised under standardised feeding conditions, and no adverse uterine effects were observed. For routine use in genetic conservation programmes, standardised extender preparation and EYP sterilisation by gamma irradiation (Pillet *et al.* 2011), would strengthen the reproducibility and biosafety of EYP-based extenders.

Sperm quality is not the sole determinant of reproductive success in donkeys. Pregnancy rates following AI with fresh semen are generally reported to be lower in jennies than in mares, regardless of the fertility of the male (Fanelli *et al.* 2023). Morphologically, the cervical lumen of the jenny is narrower and more convoluted than that of the mare, making successful intrauterine insemination more challenging (de Oliveira *et al.* 2016, Fanelli *et al.* 2023). Furthermore, advancing age and multiparity predispose the dorsal commissure of the vulva to shift above the pelvic floor, thereby increasing the risk of pneumovagina and ascending bacterial contamination. This condition promotes chronic inflammation of the reproductive tract and may result in embryonic or fetal loss if it occurs during gestation. It is also recognised as one of the most common causes of infertility in breeding females (Christoffersen & Troedsson 2017).

Transient uterine inflammation following natural mating or AI is a normal physiological response. This response results from a tightly regulated balance between multiple pro- and anti-inflammatory factors and is characterised by infiltration of polymorphonuclear neutrophils (PMNs) in the uterus to eliminate excess spermatozoa, bacteria and extender components (Miró *et al.* 2021). In jennies, the post-insemination inflammatory response is more pronounced than in mares, with large numbers of PMNs and some eosinophils in the uterine lumen 6 h after AI (Miró *et al.* 2020). According to Miró *et al.* (2013), this reaction is enhanced by the presence of extenders, biochemical alterations of the sperm membrane and the absence of SP in the insemination dose. In some mammalian species, SP removal is a prerequisite for successful sperm preservation (Brinsko *et al.* 2000, Leal *et al.* 2018; Neila-Montero *et al.* 2024). However, SP plays an important role in the uterine environment during the peri-conception period and also influences sperm motility and survival by limiting oxidative damage and sperm binding to PMNs or other phagocytic cells in the uterus (Rota *et al.* 2008, Christoffersen & Troedsson 2017, Miró *et al.* 2020, Fedorka *et al.* 2024). In this study, transrectal ultrasonography did not reveal any abnormal fluid accumulation indicative of an exacerbated inflammatory response. These findings suggest that low-volume intrauterine insemination at the tip of the uterine horn represents a promising approach in jennies, potentially limiting excessive sperm selection by the female reproductive tract, reducing the risk of post-insemination endometritis and optimising sperm deposition near the sperm reservoir and the site of fertilisation.

In conclusion, storage of semen at 4°C appears to be an effective strategy for maintaining sperm viability and fertility for up to 24 h, provided that an appropriate extender is used to protect sperm membranes from cold-shock-induced damage. Prior removal of seminal plasma by centrifugation may further improve semen preservation by limiting the deleterious effects of seminal plasma components on spermatozoa during storage. When this procedure is not feasible, supplementation of a milk protein-based extender with EYP represents a valuable alternative for maintaining sperm integrity.

Furthermore, optimisation of insemination techniques represents an additional approach for improving fertility in jennies. Deep intrauterine insemination at the tip of the uterine horn, although requiring specific technical expertise and regular monitoring of the oestrous cycle, allows substantial reductions in both sperm numbers per dose and insemination volume. This strategy may contribute to more efficient use of cooled semen and, consequently, improved reproductive management and breeding efficiency.

The birth of four Grand Noir du Berry foals provides *in vivo* validation of this approach, combining 24 h semen storage at 4°C with deep intrauterine insemination. These findings open new opportunities for assisted reproduction programmes and the preservation of genetic diversity in small donkey populations.

## Supplementary materials





## Declaration of interest

The authors declare that there is no conflict that could be perceived as compromising the impartiality of the research presented.

## Funding

A Petit is supported by a post-doctoral fellowship funded by the Centre-Val de Loire Region. This study was supported by INRAE and the Centre-Val de Loire Region (SAFRAN project).

## Author contribution statement

XD and AP conceptualised the study. AP performed the experiments, analysed the data and drafted the manuscript under the supervision of XD and FR. RE performed the proteomic analyses under the supervision of VL. FR, AG, AC, PB, TB and YG participated in semen collection, ultrasound monitoring of the jennies and AI. All authors read, contributed to and approved the final version of the manuscript.
