# Supplementary material for: Comparison of donkey and stallion semen preservation: effects of egg yolk plasma, seminal plasma and extenders on sperm motility, MALDI profiles and fertility
Source: Reprod Fertil. 2026 Sep 25;7(3):RAF260059. doi: 10.1530/RAF-26-0059 (PMC13641145; doi:10.1530/RAF-26-0059)
Supplement: Supplementary file 1 [file RAF-26-0059_supplementary_figure_1.pdf]

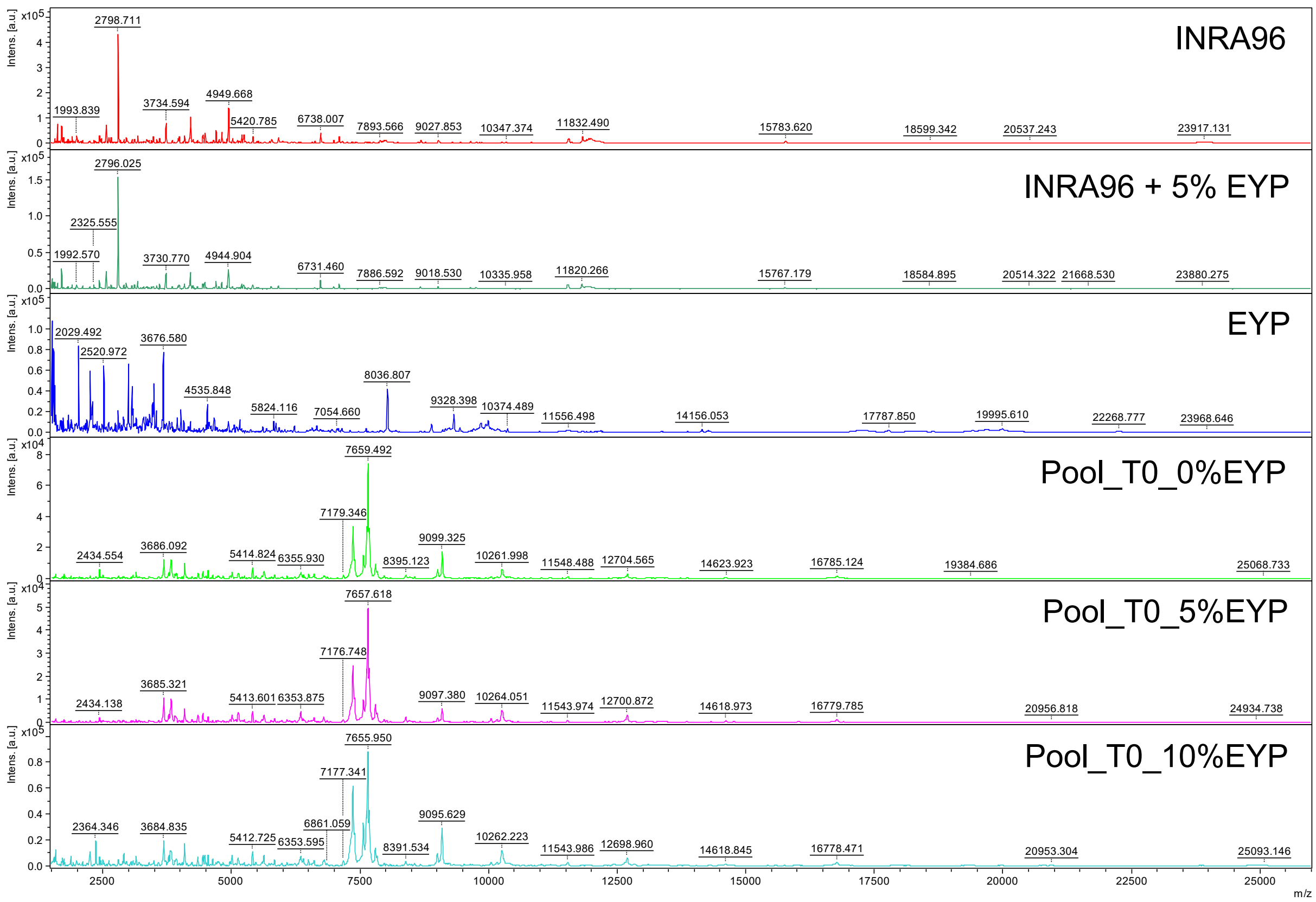

MALDI profiles of INRA96 alone, EYP alone, INRA96 supplemented with 5% EYP, and pooled semen from five jacks diluted in INRA96 supplemented with 0%, 5%, or 10% EYP, in the 1,500–26,000 m/z range.

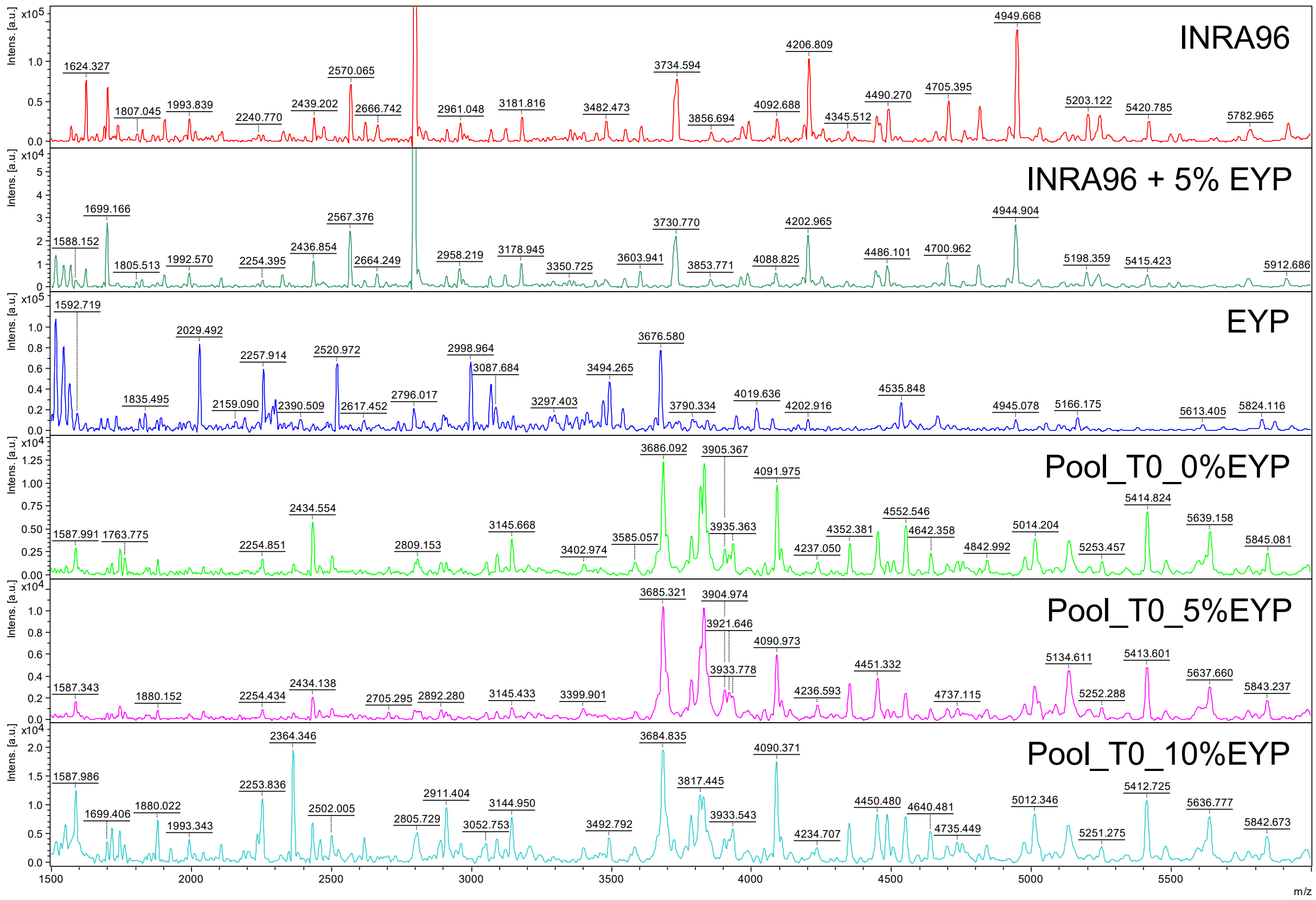

MALDI profiles of INRA96 alone, EYP alone, INRA96 supplemented with 5% EYP, and pooled semen from five jacks diluted in INRA96 supplemented with 0%, 5%, or 10% EYP, in the 1,500–6,000 m/z range.

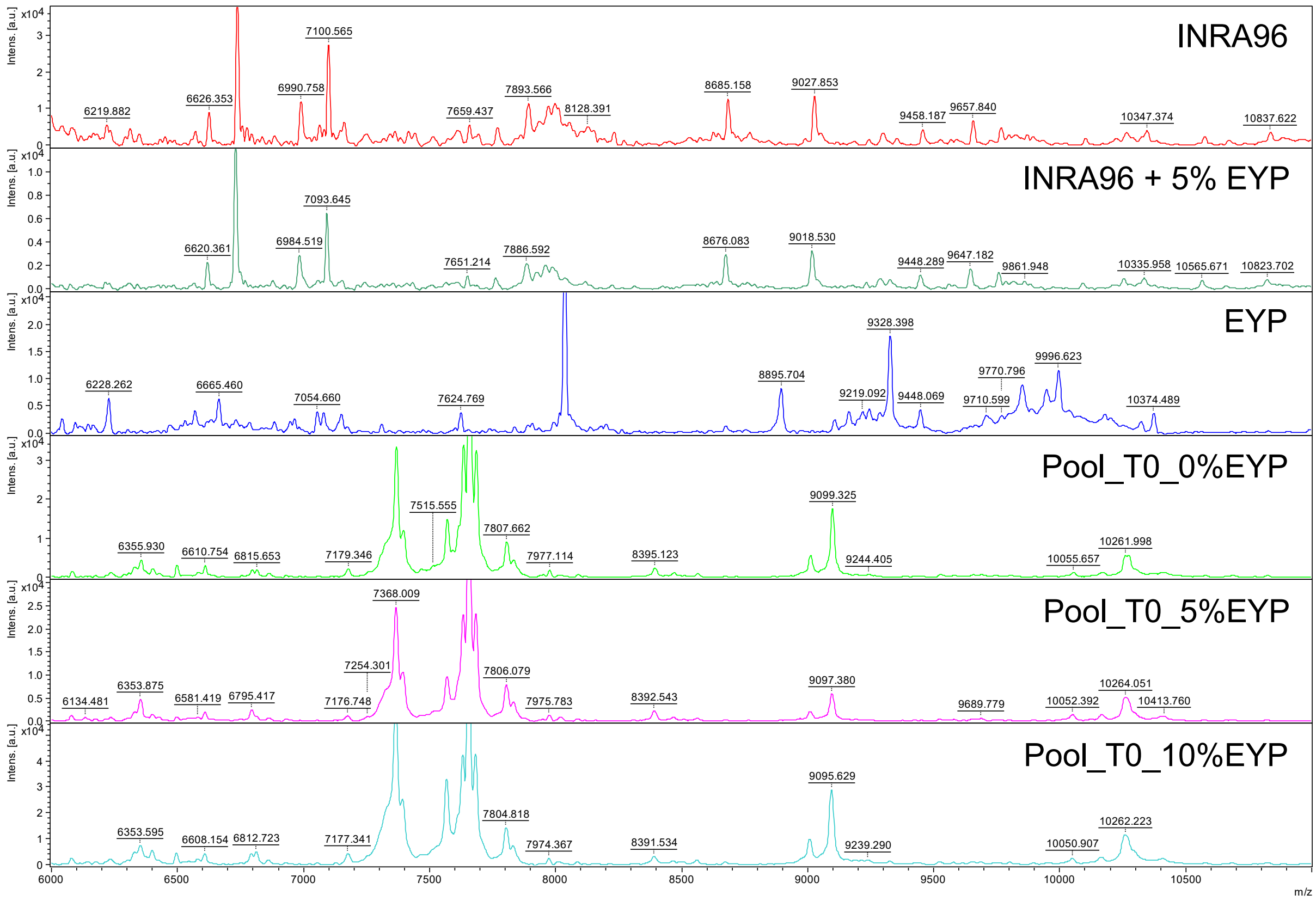

MALDI profiles of INRA96 alone, EYP alone, INRA96 supplemented with 5% EYP, and pooled semen from five jacks diluted in INRA96 supplemented with 0%, 5%, or 10% EYP, in the 6,000–11,000 m/z range.

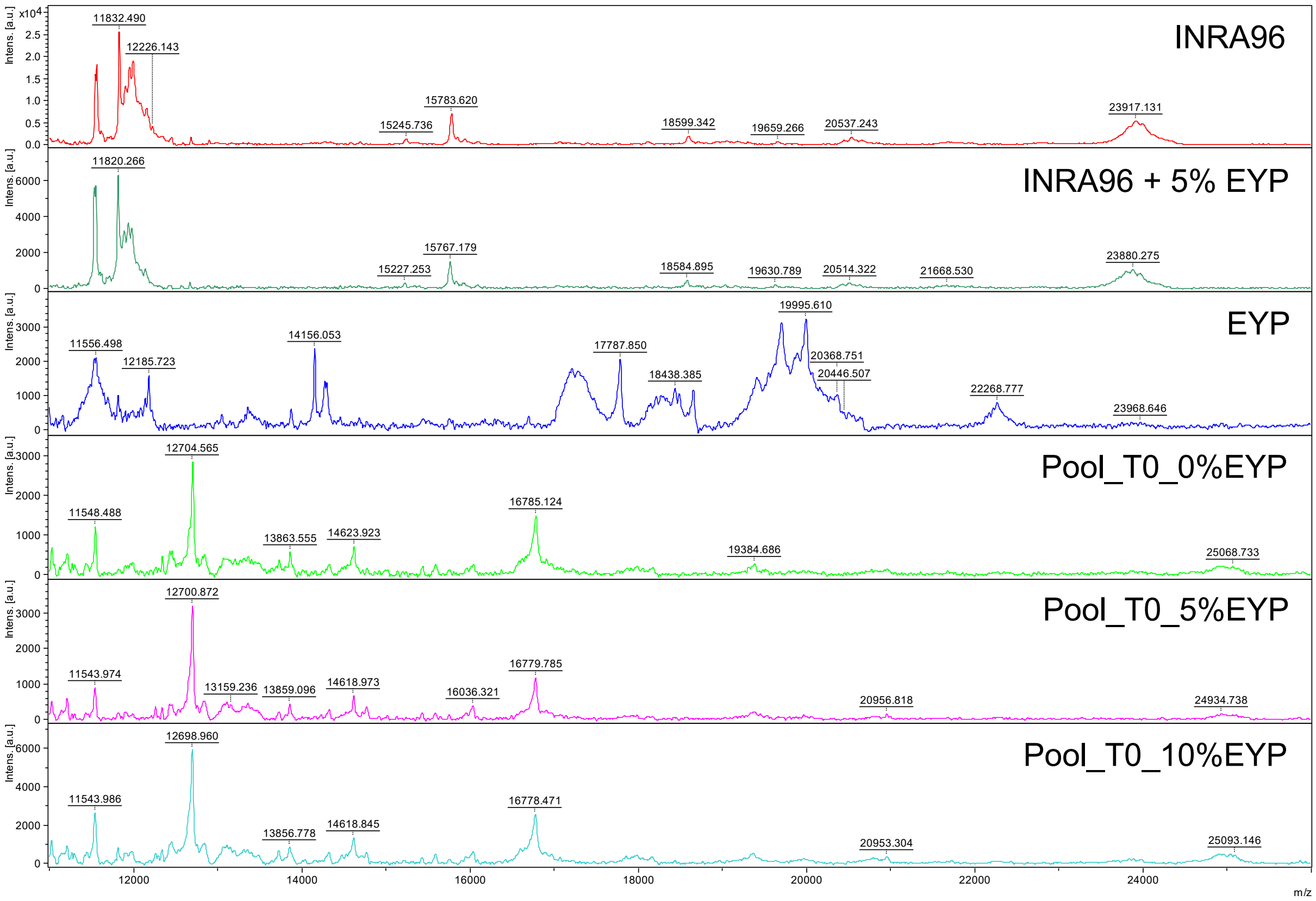

MALDI profiles of INRA96 alone, EYP alone, INRA96 supplemented with 5% EYP, and pooled semen from five jacks diluted in INRA96 supplemented with 0%, 5%, or 10% EYP, in the 11,000–26,000 m/z range.
