# Supplementary material for: Comparison of donkey and stallion semen preservation: effects of egg yolk plasma, seminal plasma and extenders on sperm motility, MALDI profiles and fertility
Source: Reprod Fertil. 2026 Sep 25;7(3):RAF260059. doi: 10.1530/RAF-26-0059 (PMC13641145; doi:10.1530/RAF-26-0059)
Supplement: Supplementary file 2 [file RAF-26-0059_supplementary_table_1.pdf]

Female	Date of birth	Age in 2025	Weight upon arrival in 2024 (Kg)	Weight in April 2025 (Kg)	Weight in October 2025 (Kg)	Number of offspring	Pneumovagina / Uterine fluid	Notes
1	27/04/2008	17	242	225	.	1	No	
2	27/06/2010	15	270	273	285	0	No	
3	03/07/2010	15	378	334	336	1	No	Presence of uterine cysts
4	08/09/2010	15	326	330	.	4	No	
5	05/07/2011	14	365	338	.	2	No	
6	31/05/2014	11	370	348	356	0	No	
7	28/05/2018	7	410	406	.	0	No	
8	15/06/2018	7	.	307	302	2	No	
9	01/01/2019	6	292	290	295	Unknown	No	
10	01/07/2019	6	316	274	269	0	No	
11	14/04/2020	5	348	351	.	0	No	
12	22/05/2020	5	310	324	338	0	No	
13	29/06/2020	5	.	266	262	0	No	
